# Hantavirus Pulmonary Syndrome Outbreak, Brazil, December 2009–January 2010

**DOI:** 10.3201/eid1911.120463

**Published:** 2013-11

**Authors:** Ana Cláudia Pereira Terças, Marina Atanaka dos Santos, Marta Gislene Pignatti, Mariano Martinez Espinosa, Alba Valéria Gomes de Melo Via, Jaqueline Aparecida Menegatti

**Affiliations:** Federal University of Mato Grosso, Cuiabá, Brazil (A.C.P. Terças, M.A. dos Santos, M.G. Pignatti, M.M. Espinosa);; University Center Cândido Rondon, Cuiabá (A.C.P. Terças);; State Secretary of Health of Mato Grosso, Cuiabá (A.V.G. de Melo Via, J.A. Menegatti)

**Keywords:** hantavirus pulmonary syndrome, outbreak, epidemiological surveillance, respiratory infections, hantavirus, viruses, Brazil

## Abstract

An outbreak of hantavirus pulmonary syndrome occurred in the Sobradinho Indian settlement of the Kayabí ethnic group in northern Mato Grosso during December 2009–January 2010. We conducted a retrospective study to clarify the outbreak’s epidemiologic and clinical characteristics. Results suggest a relationship between the outbreak and deforestation and farming expansion in indigenous areas.

Hantavirus pulmonary syndrome (HPS) was first identified in 1993 in the semi-arid southwestern region of the United States known as the Four Corners (*1*,*2*). This manifestation occurred in the form of an outbreak of the Sin Nombre virus in a community of Navajo Indians.

HPS is associated with American wild rodents of the family *Cricetidae*. Members of the the *Sigmodontinae* subfamily serve as rodent reservoirs of hantaviruses, and persons become infected mainly through inhaling rodent secretions and aerosolized excreta (*3*–*5*). Propitious ecologic conditions such as social, economic, and spatial factors facilitate the initiation and maintenance of the disease and determine its emergence (*6**–**7*).

In Brazil, areas of deforestation and environmental change, which have resulted from economic growth and agricultural production in the past 20 years, has had an effect on the number of HPS cases (*8*,*9*). In Mato Grosso, the first HPS cases were recorded in 1999 in the city of Campo Novo do Parecis, where the Castelo dos Sonhos and Laguna Negra viral strains were identified (*9*). According to the State Health Secretary of Mato Grosso, 203 cases were registered from 1999 to 2010; the death rate was 42.8%.

An outbreak of identified HPS cases occurred in the Sobradinho Indian settlement of the Kayabí ethnic group within the Xingu Indigenous Park in northern Mato Grosso during December 2009–January 2010. We conducted a retrospective study to clarify the epidemiologic and clinical characteristics of the outbreak.

## The Study

The Xingu Indigenous Park was created in 1961 and occupies 2.9 million acres of the Amazon region in the state of Mato Grosso (*10*). In 2011, 1,331 persons lived in the park in 38 Indian settlements (*11*).

**Figure 1:**
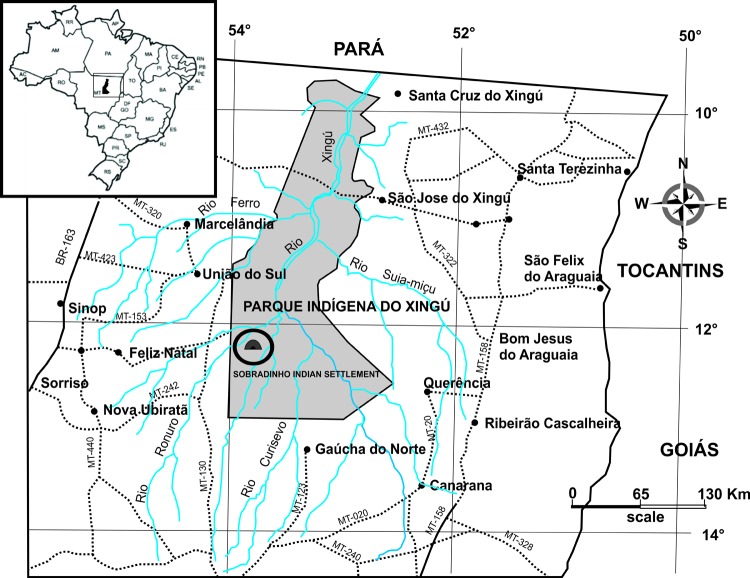
Xingu Indigenous Park and the Sobradinho Indian settlement, Mato Grosso State, Brazil.

The Sobradinho Indian settlement is located in the far northern region of Mato Grosso (11°15′30´´S, 53°44′53´´W), and the settlement is part of the Xingu Indigenous Park (Figure 1). From the 1950s onwards, the Kayabí people started moving to the Xingu Indigenous Park and now currently reside in this Indian settlement (*12*).

We analyzed documents and also examined all the notification forms of patients with confirmed cases of HPS during December 2009–January 2010 who were likely infected in the Sobradinho Indian settlement, as well as all the documented records of the epidemiologic investigation from the office of the State Health Secretary of Mato Grosso. Data from medical records were not used. The study was approved by the Committee of Ethics in Research.

Indigenous areas in Brazil were considered to be unaffected by hantavirus until the beginning of February 2010, when serologic tests were performed on blood samples from 3 patients who lived in the Sobradinho Indian settlement. We used ELISAs to test the samples for IgG with the specific antigen for Sin Nombre virus and for IgM with the Laguna Negra and Andes viruses.

The first notification that aroused suspicion of hantavirus infection occurred on January 12, 2010, in patients from the Sobradinho Indian settlement. The 33 samples collected during an epidemiologic investigation were tested for hantavirus antibodies. Of the samples, 17 (51.5%) were from inhabitants of a single home (house 3) while the outbreak was being investigated. Of the 33 samples that underwent serologic testing, 17 (51.1%) were positive for hantavirus antibodies,9 (52.9%) were positive for IgM/IgG, and 8 (47.1%) were positive only for IgG.

**Figure 2:**
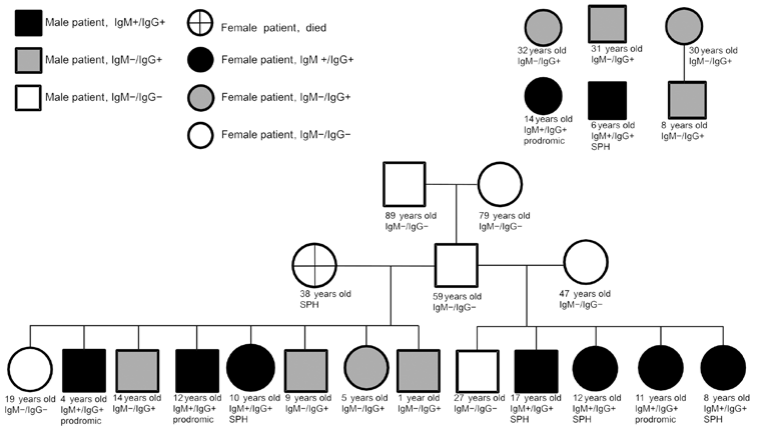
Genogram for residents of house 3 and other persons infected during the hantavirus outbreak in the Sobradinho Indian settlement, January 2010, Mato Grosso State, Brazil. HPS, hantavirus pulmonary syndrome; HCPS, hantavirus cardiopulmonary syndrome.

Of the 17 examined persons who lived in house 3, 11 (64.7%) had positive serologic test results for hantavirus and survived: 7 (41.2%) had IgM/IgG antibodies, and 4 (23.5%) had IgG antibodies. In addition, a member of this family (mother) died on January 11, 2010 (Figure 2).

The other 6 infected persons who did not live in house 3 would go to this house on a daily basis, and these persons exhibited unspecified signs and symptoms. As a consequence, they underwent serologic testing. Four tested persons were positive for IgG; 2 tested positive for IgM/IgG (Figure 2). In the family with the deceased mother, a 19-year-old girl and a 4-year-old boy were infected.

A 38-year-old woman, who lived with 7 patients with symptoms, died on January 11; she exhibited the same initial symptoms and reported insufficient breathing before her death. She received no assistance and was buried inside a hut in accordance with her cultural traditions.

Cases occurred equally in male and female patients. Patient ages ranged from 1 to 38 years, with an average of 13.7 years (Table 1). A total of 14 signs and symptoms were reported; thelargest proportions of patients experienced fever (100%), dry cough (72.2%), and abdominal pain (66.7%) (Table 2). The clinical manifestations were recorded from December 30, 2009, through 28 January 28, 2010. Thus, the interval between the cases did not exceed the disease incubation period, which may vary from 4 to 55 days (*7*).

**Table 1:** Distribution of HPS patients, according to sex, age, criterion of confirmation, hospitalization, and evolution of the cases, Sobradinho Indian settlement, Mato Grosso State, Brazil, January 2010*

Characteristic	No. (%) male	No. (%) female	No. (%) total	
Age, y				
<5	2 (22.2)	–	2 (11.1)	
5–10	3 (33.3	3 (33.3)	6 (33.3)	
11–15	3 (33.3)	3 (33.3)	6 (33.3)	
16– 20	1 (11.1)	–	1 (5.6)	
>20	–	3 (33.3)	3 (16.7)	
Criterion of confirmation				
Laboratorial	9 (100.0)	8 (88.9)	17 (94.4)	
Clinical and epidemiologic	–	1 (11.1)	1 (5.6)	
Hospitalization				
Hospital stay	3 (33.3)	3 (33.3)	6 (33.3)	
Observations at house I	2 (22.2)	3 (33.3)	5 (27.8)	
Never left Indian settlement	4 (44.4)	3 (33.3)	7 (38.9)	
Evolution				
Cure	9 (100.0)	8 (88.9)	17 (94.4)	
Death	–	1 (11.1)	1 (5.6)	
Total	9 (50.0)	9 (50.0)	18 (100.0)	

*HPS, hantavirus pulmonary syndrome; –, none. Source: Health State Secretary of Mato Grosso, 2011.

**Table 2:** Interval between symptoms, notification, collection of the first serologic tests, hospitalization and duration of hospitalizations for patients with cases of HPS in the Sobradinho Indian settlement, Mato Grosso State, Brazil, January 2010*

Interval, d	No. patients	Minimum	Median	Maximum	Average	SD	CI	CV
Between symptoms and notification	17	3	43.00	53	35.65	18.76	26.00–45.30	52.64
Between symptoms and first collection of for serologic testing	16	6	43.50	54	36.25	18.66	26.31 – 46.19	51.47
Between symptoms and hospitalization	6	3	3.00	4	3.17	0.41	2.74 – 3.60	12.89
Duration of hospitalization	6	4	4.00	10	6.00	3.10	2.75 – 9.25	51.64

*HPS, hantavirus pulmonary syndrome; CV, coefficient of variance.

During this outbreak, pulmonary disease developed in 6 patients, and 5 survived. The symptoms preceding the death of 1 patient were recorded by her husband, who drew attention to her breathing difficulty and intense sudoresis. These symptoms could have been signs of circulatory shock.

The time between the onset of the symptoms and hospitalization was, on average, 3.17 days (median3) (Table 2). The duration of hospitalization ranged from 4 to 10 days, with a median of 4 days and an average of 6.40. The death rate in this outbreak was 10% lower than the state rate (33.3%) and the national rate (44.4%) for 2010 HPS outbreaks

The hantaviruses known to circulate in this area are the strains Castelo dos Sonhos *(*in *Oligoryzomys utiaritensis*rats*)* and the Laguna Negra *(*in *Calomys aff.callosus* mice*)* (*9*,*13*–*15*). These are typically responsible for cases of HPS in Mato Grosso, in southern Pará State (Castelo dos Sonhos), and in the cities near the Xingu Indigenous Park. In the outbreak described here, no PCR or sequencing was done to confirm the strain.

In all of these cases, the home was the likely environment where infection occurred. However, other situations in which persons are at risk for infection include the following: harvesting and transportation of grains (30.0%) on plantations, house cleaning in a wilderness area (100.0%), contact with wild rodents and their excreta (100.0%), and contact with persons with HPS (90.0%).

Patients have become infected during housecleaning, when hantavirusesin rodent excreta could have been swept into the air. This supposition is supported by the fact that the infection was detected in the woman who did the cleaning and in children and adolescents who were also in the house. Other risky situations include agricultural activities, the management and storage of grains, and the direct contact with wild rodents and their excreta.

## Conclusions

Disease awareness and information campaigns targeted toward the prevention of hantaviruses in the Xingu Indigenous Park should be intensified, given the risk of the potential presence of infected rodents in other Indian settlements. As HPS has become recognized in Brazilian indigenous areas, new studies should be conducted to evaluate the serum prevalence among indigenous peoples. Such surveillance will allow identification of the possible reservoirs and the prevalence of hantaviruses in the area.
